# Psychometric properties of the cardiac depression scale in patients with coronary heart disease

**DOI:** 10.1186/1471-244X-12-216

**Published:** 2012-12-03

**Authors:** Litza A Kiropoulos, Ian Meredith, Andrew Tonkin, David Clarke, Paul Antonis, Julie Plunkett

**Affiliations:** 1 Melbourne School of Psychological Sciences, The University of Melbourne, Victoria, 3010, Australia; 2 Psychology Department, Royal Melbourne Hospital, Parkville, VIC, 3050, Australia; 3 School of Psychology and Psychiatry, Monash University, Monash Medical Centre, 246 Clayton Rd, Clayton, VIC, 3168, Australia; 4 MonashHeart, Monash Medical Centre, 246 Clayton Rd, Clayton, VIC, 3168, Australia; 5 Department of Medicine, Monash University, 246 Clayton Rd, Clayton, VIC, 3168, Australia; 6 Cardiovascular Research Unit, Department of Epidemiology and Preventive Medicine, Monash University, Alfred Hospital, Melbourne, VIC, 3004, Australia; 7 School of Psychology and Psychiatry, Monash University, Monash Medical Centre, 246 Clayton Rd, Clayton, VIC, 3168, Australia; 8 MonashHeart and Cardiovascular Research Centre, Monash Medical Centre, 246 Clayton Rd, Clayton, VIC, 3168, Australia

**Keywords:** Cardiac depression scale, Depression, Coronary heart disease, Validity, Reliability, Psychometric properties

## Abstract

**Background:**

This study examined the psychometric properties of the Cardiac Depression Scale (CDS) in a sample of coronary heart disease (CHD) patients.

**Methods:**

A total of 152 patients were diagnosed with coronary heart disease and were administered the CDS along with the Beck Depression Inventory- 2 (BDI-2) and the State Trait Anxiety Inventory (STAI) 3.5-months after cardiac hospitalization.

**Results:**

The CDS’s factorial composition in the current sample was similar to that observed in the original scale. Varimax-rotated principal-components analyses extracted six factors, corresponding to mood, anhedonia, cognition, fear, sleep and suicide. Reliability analyses yielded internal consistency α - coefficients for the six subscales ranging from 0.62 to 0.82. The CDS showed strong concurrent validity with the BDI-II (*r* = 0.64). More patients were classified as severely depressed using the CDS. Both the CDS and the BDI-2 displayed significantly strong correlations with the STAI (*r* = 0.61 and *r* = 0.64), respectively.

**Conclusions:**

These findings encourage the use of the CDS for measuring the range of depressive symptoms in those with CHD 3.5 months after cardiac hospitalization.

## Background

A high incidence of depression has been reported in those with coronary heart disease (CHD) specifically in those who have had coronary artery bypass graft surgery [1-3], those who have experienced myocardial infarction [2,4] and following angina [5,6]. One in every four people with CHD have been reported to meet diagnostic criteria for major depression [7-11]. Mild depression is also commonly found in those hospitalised with CHD and has been estimated to affect from one in four [9,11] to one in three of these individuals [12]. Depression has been found to affect the prognosis of patients with CHD even though some of these patients may not always meet the DSM-IV criteria for major depression [13]. Those with CHD who are more likely to experience mild to moderate depression, (based on a BDI score of 10 or more), have been found to have a subsequent mortality compared with patients with BDI scores less than 10 when assessed at 6 and 18 months following the event [14-16]. Similar findings have also been found in patients with angina, (who scored greater than 10 on the BDI), who were more likely to die of a cardiac event or to experience a nonfatal cardiac event within 1 year of the initial hospital admission compared to patients with a BDI score of less than 10 [6]. Both mild to moderate and severe depression at hospitalization have also been found to be strong predictors of depression 3 months after cardiac hospitalization [17]. Longitudinal studies have found that individuals with CHD who are depressed are less likely to adhere to their treatment regime and lifestyle recommendations following a cardiac event [18] and might be at higher risk of subsequent cardiac events [19]. Co-morbid depression and low social support has also been found to seriously impact prognosis with a 3 to 5 fold increase risk of death found in those with CHD who had poor social support [20].

A range of depression assessment tools have been used to measure depressive symptoms in CHD patients. These include the Beck Depression Inventory – 2 (BDI-2) [21], the Hospital Anxiety and Depression Scale (HADS) [22], the Center for Epidemiologic Studies Depression Scale (CES-D) [23] and the Cardiac Depression Scale (CDS) [24]. One criticism of the use of non disease specific tools in CHD patients is that symptoms such as fatigue may be mistaken for cardiac-related symptoms which may result in under-detection of the somatic symptoms of depression which have been attributed instead to cardiac problems [25].

The CDS has been developed as a depression scale to accurately assess depression in cardiac patients [24]. The original CDS has been shown to comprise of seven reliable and distinct components which include Mood, Anhedonia, Cognition, Fear, Sleep, Suicide and Hopelessness. However recent studies have found a six factor solution for the CDS [26,27].

The CDS has been employed to assess for a range of depressive symptoms in cardiac patients with various diagnoses and at different time points including use with ambulatory cardiac outpatients with a range of diagnoses including angina, heart failure, post-myocardial infarction, valvular heart cardiac disease and arrhythmias [24]; those with an acute coronary syndrome (due to myocardial ischaemia or infarction) who were assessed at 2-weeks post-hospital discharge [13]; and a cardiac rehabilitation population which consisted of a mixed group of cardiac patients including those who had a myocardial infarction, heart failure, coronary artery bypass graft surgery, angioplasty or a stent procedure who were assessed while undergoing cardiac rehabilitation [27].

However, further validation of the CDS in a CHD population who are medically stable and have settled into their community surroundings after cardiac hospitalization is needed given that studies have shown that around one third of those with CHD meet criteria for depression 3–4 months after a cardiac event [28]. The current paper presents the examination of the psychometric properties of the CDS as a measure of depressive symptoms in those who had CHD and were 3.5-months post cardiac hospitalisation, by investigating its factorial composition (as an index of its construct validity), its reliability and also comparing the CDS with the BDI-2 in its ability to discriminate those with mild, moderate and severe depression.

## Methods

### Participants and procedure

One hundred and fifty two outpatients with CHD who had been admitted for a cardiac procedure were recruited from two Coronary Care Units in Melbourne, Victoria. Recruitment took part between June, 2009 and September, 2011. Initial recruitment took place while the patient was still in hospital or straight after discharge. Of the 356 patients admitted to the coronary care units of two hospitals which were approached, 105 patients were not contactable and 99 did not want to participate, resulting in a final sample of 152 patients (response rate: 59%). The 152 patients who participated in this study consisted of 101 males (66.4%) and 51 females (33.6%) and ranged in age from 45 to 92 years (*M* = 70.34 years, *SD* = 8.18). Table 1 reports the characteristics of the depressed and non-depressed groups. As can be seen in Table 1, all participants had a diagnosis of CHD with 45 (29.6%) reporting angina, 42 (27.6%) treated with a coronary stent procedure, 32 (21.1%) who had a myocardial infarction, 20 (13.2%) who had coronary artery bypass graft surgery, and 13 (8.6%) who had angina. The majority of the sample was male, married, overseas born, not actively working or currently smoking at that time, and reported at least one other chronic health problem.

**Table 1:** Characteristics of depressed and non-depressed groups

**Measures**	**Total**	**Nondepressed**	**Depressed**	***P***
	**(*N*** **= 152)**	**(*N*** **= 93)**	**(*N*** **= 59)**	
Medical History				
Angina	45 (29.6%)	24 (25.8%)	21 (35.6%)	.198
Stent	42 (27.6%)	25 (26.9%)	17 (28.8%)	.795
Myocardial Infarction	32 (21.1%)	19 (20.4%)	13 (22%)	.813
CABG	20 (13.2%)	15 (16.1%)	5 (8.5%)	.174
Arrhythmia	13 (8.6%)	10 (10.8%)	3 (5.1%)	.223
Gender				.779
Male	101 (66.4%)	61 (60.4%)	40 (39.6%)	
Female	51 (33.6%)	32 (62.7%)	19 (37.3%)	
Born overseas	90 (59.2%)	46(49.5%)	44 (74.6%)	.001
Current smoker	19 (12.5%)	11 (57.9%)	8 (42.1%)	.719
Currently working	19 (12.9%)	14 (73.7%)	5 (26.3%)	.257
Married	115 (75.7%)	70 (60.9%)	45 (39.1%)	.888
Other health problem	113 (74.3%)	64 (70.3%)	49 (89.1%)	.009
Taking heart medication	121 (79.6%)	70 (78.7%)	51 (92.7%)	.025

*Note. CABG* = Coronary artery bypass grafting.

Ethics approval for this study was granted by Monash University, Southern Health and Western Health Ethics Committees. Inclusion criteria included having a diagnosis of CHD and no known psychiatric problems. During their hospital stay or within a week after their hospital discharge researchers contacted participants to describe the study and ask whether potential participants would like an explanatory statement and a consent form sent out to them. Potential participants were also asked whether it would be feasible for the researcher to re-contact them at a 3 month post discharge period to undertake a 1-hour face to face interview with them at a location of their choice. Those that agreed were sent out an information pack regarding the study and a consent form with a reply paid envelope to complete and return to the researcher prior to the interview or it was collected on the day of the interview. At the 3 month post-discharge time point, the researchers re-contacted participants by phone to organise an interview time. All questionnaires were administered by way of interview for all participants. Patients were interviewed 3-months after cardiac hospitalisation to minimise any possible influence of the hospital stay on levels of depression. Interviews took place at either the researcher’s office at Monash University or in the participant’s home.

### Measures

#### Depression

The 26-item Cardiac Depression Scale (CDS) [24] is designed to assess depression in adult cardiac populations. The CDS was developed as an alternative to more general depression scales that were considered to be too insensitive and unresponsive to depressive symptoms experienced by cardiac patients [24]. Average CDS scores have been reported in different cardiac groups with higher scores observed in those with heart failure compared with those with acute myocardial infarction [13]. All statements on the CDS are scored on a 7-point Likert-type scale ranging from ‘*strongly disagree*’ (1) to ‘*strongly agre*e’ (7). Participants are asked to rate how strongly they agree or disagree with each statement. A scale point of 4 indicates neither disagreement nor agreement with the item. Seven items are reverse-scored. Higher scores on the CDS indicate more severe depression. The CDS has fewer items that refer to somatic symptoms of depression compared to the BDI-2. The total CDS score is the sum of all items and ranges from 26 to 182. Cut-off scores to indicate mild, moderate or severe depression were not provided by the original authors for the CDS. However, a cut-off score of 90 for mild depression and 100 or above has been recommended to detect individuals with more severe depression [27], Wise et al.]. The 21-item Beck Depression Inventory- Version 2 (BDI-2) [21] is a widely used measure of psychological and physical symptoms of depression in adults. Each item consists of four statements indicating the degree of severity of the symptom. The items assess cognitive, behavioural, affective and somatic symptoms. Responses to items covered ‘*the past two weeks*, *including toda*y’. Scores range from 0 to 3 with 0 indicating absence of the symptoms and higher values indicating greater severity of the symptom. The BDI-2 score can be grouped into mild (10–19), moderate (20–25) and severe (>25) symptoms [21]. The internal reliability for the CDS and the BDI-2 in the current study indicated an α –coefficient of 0.91 for both scales.

#### Anxiety

The State-Trait Anxiety Inventory (state version) (STAI) [29] is a 20-item indictor of anxiety in adults. The scale evaluates feelings of tension, nervousness, worry and apprehension ‘*in the past two weeks*, *including today*’. Responses are on a 4-point Likert scale ranging from (0) ‘*not at all*’ and (3) ‘*very much so*’ with higher scores reflecting higher severity. In the current sample, the STAI showed excellent reliability (α = 0.93).

#### Quality of Life

The 26-item World Health Organisation Quality of Life scale (Brief version) (WHOQOL-BREF) [30] was used to measure Quality of Life. The WHOQOL-BREF asks about an individual’s quality of life in four domains including physical health, psychological health, social relationships and environment. Higher scores denote higher quality of life. The WHOQOL-BREF also contains one question about an individual’s perception of overall quality of life and a question about their perception of their overall health. The internal reliability for the four domains of the scale indicated alpha coefficients ranging from 0.81 to 0.85.

#### Social Support

The 12-item Perceived Social Support Scale (PSSS) [31] assesses perceived social support from family, friends and others. Higher scores indicated higher perceived social support. The internal consistency for this scale in this sample indicated an alpha coefficient of 0.92. The 7-item Enriched Social Support Instrument (ESSI) [32] measures functional social support particularly emotional support in cardiac patients. Higher scores indicate higher levels of social support. The ESSI has been previously used to examine level of social support and to assess changes in patients’ social support after cardiac treatment. The alpha coefficient for the ESSI in the current sample was 0.86.

## Results

### Factor Analysis

In order to reproduce the factor structure reported previously we undertook the factor analysis procedures used by Hare and Davis (1996) [24] and Wise, Harris and Carter (2006) [27]. Using the principal component extraction method with an eigenvalue > 1, the first-order factor analysis extracted six factors instead of the seven reported in the original study by Hare and Davis (1996) [24]. These six factors accounted for 63.14% of the variance. Further varimax rotation testing was performed and the individual items in relation to the six-factor solution were reproduced using a loading criterion of 0.40 as a cut-off point. Table 2 reports the factors extracted from the CDS by principal components analysis with varimax rotation and the Cronbach alpha’s for each factor. As can be seen, factor loadings ranged from 0.41 to 0.83. The individual items in relation to the subscales Mood, Anhedonia, Cognition, Fear, Sleep and Suicide subscales were observed to load onto factors 1, 2, 3, 4, 5 and 6 respectively. Items on the fear factor found in previous studies loaded on the Mood and Suicide factors. Similarly, items on the Anhedonia factor found in previous studies loaded on the Mood and Cognition factors.

**Table 2:** Factors extracted from the CDS by principal components analysis with varimax rotation (*N* = 152)

**Item number**	**Items**	**Mood**	**Anhedonia**	**Cognition**	**Fear**	**Sleep**	**Suicide**
13	The possibility of sudden death worries me (fear)	**.554**			.031		
18	Things which i regret most about my life are bothering me (mood)	**.592**					
21	I become tearful more easily than before (mood)	**.812**					
22	I seem to get more easily irritated by others than before (anhedonia)	**.826**	.060				
24	I lose my temper more easily nowadays (mood)	**.659**					
25	I feel frustrated (mood)	**.580**					
4R	I get pleasure from life at present (anhedonia)		**.568**				
23R	I feel independent and control in my life (anhedonia)		**.487**				
1	I have dropped many of my interests and activities (anhedonia)		**.593**				
3	I can’t be bothered doing anything much (anhedonia)		**.551**				
8	I am not the person i used to be (anhedonia)		**.409**				
16	I get hardly anything done (anhedonia)		**.412**				
15R	My mind is as fast and alert as always (cognition)			**.727**			
19R	I gain as much pleasure from leisure activities as i used to (anhedonia)		.312	**.620**			
20R	My memory is as good as it always was (cognition)			**.675**			
2R	My concentration is as good as it ever was (cognition)			**.686**			
5	I am concerned about the uncertainty of my health (fear)				**.564**		
17	My problems are not yet over (fear)				**.651**		
6	I may not recover properly (fear)				**.781**		
7	My sleep is restless and disturbed (sleep)					**.635**	
9	I wake up in the early hours of the morning and cannot get back to sleep (sleep)					**.754**	
12R	I feel in good spirits (suicide)						**.577**
26	I am concerned about my capacity for sexual activity (fear)				.248		**.573**
10	I feel like im living on borrowed time (fear)				.183		**.433**
11	Dying is the best solution for me (suicide)		.747				**.139**
14	There is only misery in the future for me (suicide)		.663				**.252**
**Cronbach’s alpha**		**.77**	**.82**	**.66**	**.74**	**.80**	**.62**

### Reliability analysis

The total 26-item CDS scale showed good reliability (α = 0.91). As seen in Table 2, the Cronbach alpha coefficients for the six CDS subscales were acceptable and ranged from 0.62 (Factor 6: Suicide) to 0.82 (Factor 2: Anhedonia).

### Concurrent validity

Pearson’s correlation coefficients were used to established the CDS’s concurrent validity with a moderate to high correlation between the relevant scales deemed as acceptable (*r* > = 0.3). A significant moderate to high correlation between the CDS and the BDI-2 scale (*r* = 0.65, *p* < 0.001) and between the BDI-2 and the STAI (*r* = 0.64, *p* < 0.001) was observed. Similarly, moderate to high correlations were found between the CDS and the STAI scale (*r* = 0.61, *p* < 0.001), the CDS and the physical QOL subdomain (*r* = −0.64, *p* < 0.001), psychological QOL subdomain (*r* = −0.60, *p* < 0.001), social QOL subdomain (*r* = −0.46, *p* < 0.001) and environmental QOL subdomain (*r* = −0.43, *p* < 0.001), and the PSSS (*r* = −0.42, *p* < 0.001). A weaker correlation was found between the CDS and the ESSI, (*r* = 0.18, *p* < 0.05).

### Relationships between the CDS and the BDI-2

Analyses of variance were used to determine the differences between the depressed and the non-depressed groups on the depression, anxiety, quality of life and social support measures. Based on previous research, inclusion in the depressed group in the current study was determined by a BDI-2 score of > = 10 [13]. Results of the analyses of variance can be seen in Table 3. Participants in the depressed group were more likely to score significantly higher on the CDS, BDI-2, STAI and lower on the QOL physical, psychological, social and environment subdomains, and on the ESSI and PSSS compared to the non-depressed group. The recommended cut-offs for the CDS and the BDI-2 employed in previous studies were used in the current study to examine how many outpatients were classified in the mild, moderate and severe depression groups [13,26,27]. Specifically, BDI-2 cut-off score of 10 for mild-moderate depression and 19 for severe depression and CDS cut-off score of 90 for mild to moderate depression and 100 for severe depression were used. The percentages of patients with mild to moderate and severe depression according to the CDS and the BDI-2 are presented in Table 4. As can seen, the CDS classified 72 (47.4%) of the participants as not depressed compared to 93 (61.2%) of the participants by the BDI-2 scale. Similar results for the two scales were found when classifying the mild to moderately depressed participants in the sample. Specifically, the CDS classified 38 (25%) of the participants as mild to moderately depressed compared to the BDI-2 which classified 37 (24.3%) of the participants. However, it seemed that the CDS classified more participants as severely depressed in the current sample compared to the BDI-2, with the CDS classifying 42 (27.6%) of the participants as severely depressed whereas the BDI-2 classified only 22 (14.5%) of participants as such.

**Table 3:** Summary of ANOVA results for depressed and non-depressed groups

	**Total sample (*n*** **= 152)**	**Depressed mean**^**a**^**(SD) (*n*** **= 59)**	**Non-depressed mean (SD) (*n*** **= 93)**	***F***	***P***	***η***^***2***^
BDI-II	9.43 (8.92)	17.81 (8.68)	4.11 (3.05)			
CDS	89.30 (22.72)	103.38 (15.88)	80.37 (21.92)	48.71	< 0.001	.25
STAI	39.27 (12.98)	48.06 (11.81)	33.57 (10.27)	63.26	<0.001	.30
QOL 1	12.97 (3.29)	10.65 (2.80)	14.45 (2.68)	68.57	<0.001	.32
QOL 2	14.40 (3.18)	12.28 (2.65)	15.73 (2.74)	57.54	<0.001	.28
QOL 3	14.95 (3.68)	13.20 (3.48)	16.05 (3.38)	24.60	<0.001	.14
QOL 4	15.42 (2.71)	14.01 (2.20)	16.31 (2.63)	30.80	<0.001	.17
ESSI	27.50 (5.90)	26.24 (6.36)	28.31 (5.47)	4.43	.037	.03
PSSS	66.49 (14.75)	61.10 (14.04)	69.91 (14.22)	13.50	<0.001	.09

*Note.*^a^ = *BDI-II* > = 10. *CDS* = Cardiac Depression Scale; *BDI-II* = Beck Depression Inventory-2; *STAI* = State Trait Anxiety Inventory; *QOL 1* = Quality of life physical subdomain; *QOL 2* = Quality of life psychological subdomain; *QOL 3* = Quality of life social relationships subdomain; *QOL 4* = Quality of life environment subdomain; *ESSI* = ENRICHD Social Support Instrument; *PSSS* = Perceived Social Support Scale.

**Table 4:** Percentages of patients with mild, moderate and severe depression according to the CDS and the BDI-2

**Not depressed**		**Mild/moderate depression**		**Severe depression**	
CDS < 90	BDI-2 < 10	CDS > = 90	BDI-2 (10–18)	CDS > = 100	BDI-2 (19+)
(*N*/%)	(*N*/%)	(*N*/%)	(*N*/%)	(*N*/%)	(*N*/%)
72 (47.4%)	93 (61.2%)	38 (25%)	37 (24.3%)	42 (27.6%)	22 (14.5%)

*Note. N* = 152; *CDS* = Cardiac Depression Scale; BDI-2 = Beck Depression Inventory-2.

## Discussion

The aim of this study was to examine the psychometric properties of the CDS and provide further data to support the use of the CDS in a CHD population who have been medically stable and living in their usual community environment 3.5 months after cardiac hospitalisation.

Consistent with previous research examining the construct validity of the CDS in a cardiac population [26,27] factor analysis of the CDS yielded six factors rather than the seven factor solution reported by the authors of the scale [24]. Four of these factors were almost identical to the Mood, Anhedonia, Cognition and Sleep Disturbance factors reported originally [24] and by other researchers [26,27]. Similar to previous research [24,27], all factors except Suicidal Ideation demonstrated acceptable reliability and a potential explanation for this may be the low number of items comprising this factor [27].

There is growing evidence that the CDS is characterized by adequate psychometric properties when applied to cardiac samples. Reliability has been found to be high across cardiac samples when assessed in terms of internal consistency (coefficient alphas have ranged from 0.88 to 0.92). Moreover, the validity of the CDS has been used successfully to discriminate between cardiac patients diagnosed as depressed vs. nondepressed. The CDS has also been found to have good concurrent validity with the BDI-2 (ranging from 0.69 to 0.73) and the STAI (*r* = 0.80) [13,26,27]. Consistent with this, the current study also found that the CDS and the BDI-2 demonstrated good concurrent validity as seen in the significant correlations between the CDS and the BDI-2 and the anxiety, quality-of-life and social support measures [13,24,27]. The correlations between the CDS, BDI-2 and the STAI were within the ranges found in previous research examining the psychometric properties of the CDS [13]. The moderate to strong correlation between the CDS and the BDI-2 scores for the current sample indicating a moderate concurrent validity suggests that the two scales are comparable. Both the BDI-2 and CDS correlated moderately to strongly with the STAI. Moderate to strong correlations obtained between depression and anxiety measures have typically been found in cardiac populations and may also be indicative of the co-morbid depressive and anxiety symptoms experienced by these populations [13]. The internal consistency of the CDS scores in the current study was compatible with the values obtained by the original authors and other researchers.

It should be noted that the current sample of CHD patients reported a high CDS mean score compared to previous cardiac samples [13]. This may be partly explained by the high proportion of the current sample being overseas-born who have been previously found to have higher levels of depression and anxiety [33] which could have contributed to the higher mean depression score. It is possible that depression could have preceded the development of CHD in these participants.

In the current study, depressed participants were determined by a BDI-2 score of 10 or more. Both the CDS and the BDI-2 identified that almost one in four patients (25% vs. 24.3%) had symptoms indicative of mild to moderate depression. These results are comparable to those reported by Wise, Harris and Carter (2006) [27] who found that the CDS classified 17% of their cardiac outpatient sample as mild to moderately depressed and other studies which found mild to moderate depression in 17-30% of cardiac patients [10,34]

While the BDI-2 suggested that 14.5% had symptoms indicative of severe depression, the CDS indicated that in the same cohort, 27.6% of patients were suffering severe depression which is almost one in three patients as opposed to almost one in six by the BDI-2. This discrepancy may be due to the CDS items detecting more severe symptomatology compared to the BDI-2, the cut-off score used for detecting severe depression with the CDS and the possibility that the CDS may be overdiagnosing severe depression in this sample. However, these results are comparable to previous research which found that the CDS classified 21-25% of cardiac outpatients as severely depressed [10,11,15,18,27] and that around one-third of cardiac patients meet criteria for depression 3 to 4 months after a cardiac event [28].

### Limitations and future directions

A major limitation of the current study is that no diagnostic interviews to assess for Major Depressive Disorder or Episode were concurrently undertaken with participants to validate the levels of depression found with the CDS and the BDI-II. The current study was not able to evaluate the sensitivity or specificity of the CDS in a fully meaningful way. Further research employing larger sample sizes is required to determine whether the CDS is a more sensitive measure of depression in the 3–4 month time period after a cardiac event or whether the CDS is yielding more false positives. The current results need to be replicated given that the current sample was less than what has been recommended for conducting factor analysis with suggestions that a minimum sample size of 200 be used [35]. Future research should also examine the CDS with other screening measures for depression and anxiety that have fewer somatic items such as the Hospital Anxiety and Depression Scale (HADS).

Another limitation is that no information was gathered from those that declined to participate and no comparison can be made between this sample and those that agreed to participate in the current study.

Effective screening after a cardiac event and procedure has implications for the effective management of depression to assist cardiac patients in their therapeutic compliance and in their prognosis. Given that those in the current sample who were depressed also scored lower on the social support and quality of life measures, overall treatment approaches should focus on strategies to increase social supports and quality of life in these patients.

## Conclusions

Depression has been shown to influence health outcomes in those with CHD. Accurate and timely assessment of depression in CHD patients has the potential to influence prognosis and reduce suffering in those who have been discharged after cardiac hospitalization. The current study has shown that the CDS can be used to screen and detect the range of depressive symptomatology, ranging from mild to severe, in CHD patients who are medically stable and settled in their community surroundings 3.5 months following discharge after cardiac hospitalization.

## Competing interests

The authors declare that they have no competing interests.

## Authors’ contributions

LK contributed to the conceptualisation and design of the research, the data collection, analysis and interpretation of the study results and in the writing of the manuscript. IM, AT and DC contributed to the design of the study, contributed their clinical and research expertise in the areas of assessment, depression and CHD and provided assistance in the interpretation, translation and write up of the findings. PA and JP contributed to design and data collection. All authors approved the final manuscript.

## Pre-publication history

The pre-publication history for this paper can be accessed here:

http://www.biomedcentral.com/1471-244X/12/216/prepub
